# Organic waste flow as an ASF vulnerability: a multi-sector participatory systems workshop in eastern Dominican Republic

**DOI:** 10.3389/fvets.2026.1845550

**Published:** 2026-06-03

**Authors:** Yussaira Castillo-Fortuna, Wendy González, Luis Pablo Hervé-Claude, José Lizardo-Núñez, Lilianny Sosa-Márquez, Julia-Esther Roa, Raysa Reyes-Santiago, Juan Pablo Villanueva-Cabezas

**Affiliations:** 1 Instituto de Enfermedades Infecciosas y Zoonóticas, Universidad Autónoma de Santo Domingo (UASD), Santo Domingo, Dominican Republic; 2 Escuela de Medicina Veterinaria, Universidad Autónoma de Santo Domingo (UASD), Santo Domingo, Dominican Republic; 3 Dirección General de Ganadería (DIGEGA), Santo Domingo, Dominican Republic; 4 Lewyt College of Veterinary Medicine, Long Island University, Brookville, NY, United States; 5 Facultad de Medicina Veterinaria, Universidad San Sebastián, Concepción, Chile

**Keywords:** Caribbean region, disease introduction risk, flow networks, participatory mapping, transboundary animal diseases, transdisciplinary co-design, interdisciplinary research

## Abstract

**Introduction:**

Following its confirmation in 2021, African Swine Fever (ASF) has been endemic on the island of La Hispaniola since 2023, remaining the only active outbreak in the Americas. Several backyard pig producers in the eastern Dominican Republic depend on organic food waste from a major Caribbean tourism region to feed pigs, creating a complex risk network amongst waste generators, collectors, and pig producers. A participatory systems approach was needed to map this network, identify vulnerabilities, and develop mitigation strategies.

**Methods:**

A two-day workshop in Santo Domingo (February 24–25, 2026), as part of the PROCINORTE project, brought together hotel representatives, councils, the Ministry of Environment, DIGEGA, pig producers, and academics. The workshop framed ASF as a systemic vulnerability of the organic waste chain. Day 1 covered ASFV epidemiology, swill transmission, Caribbean practises, and Puerto Rico’s regulations around thermal treatment of swill, followed by system mapping and focus groups. Day 2 used scenarios, STEEP analysis, and an Impact/Effort Matrix to prioritise recommendations.

**Results:**

Mapping revealed informal waste chains with poor traceability, the general absence of thermal treatment, and shared collection vehicles as transmission risks. STEEP analysis identified barriers: cultural reliance on untreated waste, existing but underused thermal technology, disproportionate costs to producers of regulating organic waste processing, potential environmental benefits, and regulatory delays. The Impact/Effort Matrix prioritised the enforcement of regulations governing landfill and animal access, whilst infrastructure improvements for small producers require significant investment and reform.

**Discussion:**

ASF biosecurity requires coordinated One Health governance across tourism, waste management, and pig farming. Enforcing existing regulations is an immediate step. The workshop’s approach provides a replicable model for participatory ASF risk governance in the Caribbean.

## Introduction

African Swine Fever (ASF) is an important animal transboundary disease that was first diagnosed in Africa and has now spread to all continents in the world, including Africa, Asia, Europe and the Americas (1). Its causal agent is the ASF virus (ASFv), a virus highly stable in suitable organic material (2) that can survive in the environment for long periods, thereby facilitating disease spread (3). The ASFv can spread through direct contact between animals, fomites, and vectors (some tick species). The disease affects only suids (e.g., domestic pigs and wild boars) and produces various clinical signs, most notably a haemorrhagic fever with high mortality (1). As of 2026, there are no commercially available vaccines for ASF in the Dominican Republic (4). Although not a zoonosis, ASF causes major economic disruptions and affects the livelihoods of farmers and consumers alike, undermining the viability of agricultural systems and creating significant trade disruptions as markets react to the disease by restricting and banning trade from affected countries (5). To date, the impact of this panzootic has been difficult to quantify, but some conservative estimates put the cost at 200 billion USD in China in 2018–2019 (6) and 573 million USD in Vietnam only by December 2019 (7).

The Dominican Republic had been free from ASF for nearly 40 years when two outbreaks were confirmed in July 2021 in the provinces of Montecristi and Sánchez Ramírez, caused by a genotype II strain identical to those circulating in Russia and Eastern Europe (8). By September 2021, 165 outbreaks had been confirmed, and 98% occurred in backyard and subsistence farms. The hypothesised entry routes included the introduction of illegal pork products, incursions from Haiti, and the feeding of untreated infectious waste to pigs (8). The Ornithodoros soft tick species (*O. moubata* group in Africa and *O. erraticus* in Europe/Portugal) recognised as major vectors for the transmission and maintenance of African Swine Fever (ASF) are not present in the Dominican Republic. Although previous studies indicated that *Ornithodoros puertoricensis,* a soft tick native to the Caribbean, could act as a vector for the ASF virus under experimental or theoretical conditions, there is currently no evidence confirming its role in the country following the 2021 reintroduction of ASF (9, 10). To date, local tick populations have not been linked to the epidemiology or persistence of the disease in the Dominican Republic.

The swine sector is economically vital to the country, generating approximately 58,000 jobs and USD 500 million in investments (11). Epidemiological assessments from November 2022 to June 2023 confirm that ASF is now endemic in the DR, with the farm-level reproductive ratio (R_0_) approaching 1 and outbreaks concentrating in backyard farms in central regions (11, 12).

ASF has significantly affected the swine sector in the Dominican Republic, estimated in 13,000 producers primarily operating informal backyard farms (13). After the July 2021 re-emergence of African Swine Fever (ASF) in the Dominican Republic, failures in transport controls, particularly the movement of infected pigs, pork products, and contaminated vehicles, were the primary contributors to the virus’s spread. Despite official bans, informal animal transportation and inadequate disinfection protocols further accelerated the outbreak (14).

Swill feeding, which involves giving pigs kitchen, restaurant, or food-industry waste without prior heat treatment, is one of the most well-documented ways ASF is introduced globally. ASFv can survive over 100 days in uncooked meat and more than 1,000 days in frozen products (15). In the Dominican Republic, swill feeding is reported exclusively by backyard farmers. Research into the pig value chain showed that these producers often take pigs to scavenge at open landfill sites (*vertederos*), creating uncontrolled secondary exposure points (11, 16). The eastern part of the Dominican Republic combines the country’s largest international tourism sector, which produces most of the organic food waste in the area, with numerous smallholder farms that may access this waste as feed. However, this interface remains entirely uncharacterised with respect to waste volume, flow routes, and biosecurity measures (15, 16).

This manuscript reports a two-day participatory multi-sector stakeholder workshop conducted to characterise the organic-waste–swill–pig system in eastern Dominican Republic, identify vulnerabilities of the system to ASF, and to co-prioritise feasible interventions to reduce the risk of ASF circulation in the system through scenario discussion, STEEP analysis and an impact/effort matrix.

## Methods

### Workshop setting

The workshop was held on 24–25 February 2026 in Santo Domingo, Dominican Republic, as part of the project “Evaluación del Riesgo de la Alimentación de Cerdos con Desperdicios en la Región este de la República Dominicana”, led by the Faculty of Agronomic and Veterinary Sciences of the Universidad Autónoma de Santo Domingo (FCAV–UASD) with financial support from IICA–PROCINORTE and USDA–APHIS. The eastern region was selected as the study context because it simultaneously hosts a large international tourism and hotel sector that generates large volumes of organic waste, a heterogeneous network of organic-waste collectors, and smallholder pig production units that rely heavily on food waste as feed. This combination creates a system in which organic waste flows directly from tourism establishments into pig production with limited oversight, making it a priority setting for assessing ASF risk at the organic waste–swill–pig interface.

### Workshop design

We used a qualitative, participatory system-assessment design. The workshop was structured as a primary data collection instrument, in which facilitated exercises generated documented outputs including system maps, stakeholder categorisations, scenario tables, STEEP matrices, and impact/effort matrices, that were treated as data for subsequent qualitative analysis. The overarching methodological premise was that ASF risk at the organic waste–pig interface is an emergent property of a multisectoral system, and that understanding and prioritising interventions require the simultaneous perspectives of all actors involved (17). The central question of the workshop was: *How does the organic-waste flow system operate in the eastern Dominican Republic, and what makes it resilient or vulnerable to ASF?* To fully explore the central question, four guiding questions were permanently displayed on a flipchart throughout both days: *What is each actor’s role in the system? How is organic waste managed? What regulations exist, and how do they function in practise?* And *how does ASF entry make the system more vulnerable for everyone?* This framing deliberately positioned ASF as a vulnerability of the system rather than the fault or responsibility of any single actor. The workshop focused on system description (Day 1) before transitioning to option building and prioritisation (Day 2). All plenary and table sessions followed *Chatham House rules* (18), and a dedicated framing session clarified that the workshop was a co-design exercise rather than an inspection or enforcement event, with explicit assurances from government and regulatory representatives to that effect. This framing aimed to reduce defensiveness and protect more vulnerable participants, particularly smallholder producers, throughout discussions. Three designated documenters captured all session outputs in written minutes, and all flipcharts, system maps and matrices were photographed at the end of each exercise.

### Participants

Participants were identified by the FCAV–UASD project team through purposive sampling, targeting individuals with direct operational knowledge of or regulatory responsibility over organic waste flows, waste use in pig production, or animal health governance in the eastern Dominican Republic. Five sectors were represented: (1) national animal health services (DIGEGA); (2) smallholder pig producers (systems up to 50 animals; Resolucion-No.-RES-MARD-2024-3, 2024); (3) the hotel sector, including waste sustainability managers; (4) municipal government and regulatory bodies (19); and (5) academia, veterinary schools and international cooperation (UASD, Universidad Central del Este, IICA–PROCINORTE, USDA–APHIS). Waste collectors were invited to participate but declined. Two international veterinary epidemiologists experienced in the design and facilitation of workshops served as the lead designer and facilitator (Villanueva-Cabezas) and co-facilitator (Hervé-Claude). Participants were seated in two heterogeneous tables from the start to ensure cross-sector interaction throughout all exercises. The group consisted of eight members from the national veterinary services, including four field veterinarians and four epidemiologists (*n* = 8), two smallholders (*n* = 2), two representatives from hotel chains (*n* = 2), two representatives from the urban solid waste department of the League of Municipalities (*n* = 2), one representative from the Ministry of the Environment’s landfill inspection department, five members from the country’s veterinary faculties—including veterinary regents, microbiologists, and public health specialists—and three representatives from international organisations engaged in the control and eradication of African swine fever in the Dominican Republic.

### Day 1: understanding the system

We opened with a technical session featuring three presentations to set the scientific context. First, the ASF situation globally and in the Caribbean, including the economic effects of the 2021 outbreak in the Dominican Republic; second, a study on knowledge, attitudes, and practises towards ASF amongst pig producers across five West Indian islands (20), which served as a reference for regional trends; and third, Puerto Rico’s successful regulatory and operational experience, where a licenced swill feeding programme mandates heat treatment of all food waste and regular certification of farms by USDA-APHIS. With this context, participants were asked to share what they or their sector would lose if ASF were permanently established in the eastern region (“shared stakes in the system*”*). This exercise helped build common ground before moving into potentially sensitive discussions and clarified the economic consequences of inaction for everyone (21).

The central exercise of Day 1 was a participatory system-mapping activity conducted at mixed-sector tables. Participatory systems mapping is increasingly used in health and One Health contexts to externalise complex knowledge held by multiple actors, identify system components and their interactions, and surface vulnerability points that are not visible from any single institutional perspective (17, 22). Each table was given a blank flipchart and asked to collaboratively draw the flow of organic waste in the eastern region, from origin (hotels) through collection and transport to final destinations (including piggeries), via formal and informal routes. Structured facilitation questions progressively deepened the maps: first, exploring who the actors are, their economic relationships, and the direction of payment within the chain; then, probing for informal, undocumented, or off-contract flows; and finally, exploring what happens to waste that is rejected by farms or produced in excess of farm absorption capacity. Once maps were considered complete, participants used green stickers to mark elements they considered functioning well and red stickers to mark friction points, bottlenecks, risks, or tensions. This sticker-based annotation draws on participatory appraisal traditions (23) for identifying perceived system vulnerabilities and areas of consensus across heterogeneous groups. Tables then presented their maps in a plenary session, during which the lead facilitator helped participants identify convergent nodes, key connectors and transversal themes emerging across all maps.

The final session of Day 1 separated participants by sector to allow sector-specific discussion before reconvening in a mixed format. The government and normative group, facilitated by a local veterinary epidemiology expert (W. Gonzalez), explored existing regulations, enforcement mechanisms and their limitations, inter-institutional coordination, and which changes were considered politically and operationally feasible in the near term. The productive chain group, facilitated by Herve-Claude and comprising hotel representatives and producers, explored daily operational practises, informal criteria and agreements on acceptable waste types, current knowledge and use of heat treatment, the economic feasibility of safer practises, and openness to incentive- or certification-based approaches. Each group was guided by structured questions designed to elicit both current practises and perceived barriers to change, consistent with established focus group methodology in veterinary and One Health research (24). Group outputs were shared in a brief closing plenary.

### Day 2: building options

Building upon key themes and system maps from Day 1, we presented three scenarios in plenary: Scenario 1 (no change—the system as currently mapped); Scenario 2 (low-cost operational improvements achievable without new regulation); and Scenario 3 (a traceable, certified organic-waste chain with potential economic incentives for compliant actors). Scenario-based approaches are used in participatory risk governance to help stakeholders reason about incremental and transformative change without being constrained by current institutional positions (25). For each scenario, the group jointly completed a structured table assessing what each sector would gain, lose and need to change, and how this influenced the identified friction points (Day 1), producing a collective “map of interests by scenario.” Mixed-sector tables then applied a STEEP framework (26)—Social, Technological, Economic, Environmental and Political-legal—to analyse why the changes required to move from Scenario 1 towards Scenarios 2 or 3. Each table identified its single most fundamental barrier and what would be required to begin removing it; findings were consolidated in plenary by STEEP dimension. Finally, an impact/effort matrix was used to prioritise proposed interventions (27). Actions drawn from the scenarios, STEEP analysis and open discussion were assigned to cards and positioned on a two-dimensional grid of expected impact on ASF risk mitigation versus effort required from the responsible sector. The workshop closed with a reflective plenary in which participants summarised their conclusions and identified what critical questions remained unresolved.

### Data analysis

The note corpus was analysed using narrative analysis. For the system mapping outputs, content was coded to identify actors (nodes), waste flows (edges), economic relationships, and vulnerability points (red stickers / friction points). For focus-group outputs, codes were organised deductively around three themes—current practises, regulatory and enforcement gaps, and barriers and enablers of change—with inductive sub-codes emerging from the content of each group. For the scenario tables, STEEP matrices and impact–effort matrix outputs, actions and barriers were classified by sector, STEEP dimension and matrix quadrant, respectively. Final counts of actions per quadrant and per enabling mechanism were generated to support a semi-quantitative description of prioritised interventions.

### Ethical considerations

The workshop was approved by the Research Department of the Vice-Rectorate for Research and Postgraduate Studies at the Autonomous University of Santo Domingo (UASD). All discussions were conducted under Chatham House rules, and participants were explicitly informed at the start of Day 1 that no statement would be attributed to a named individual or institution in any subsequent output. No individual data were collected, and no personally identifiable information appears in any document.

## Results

### Perceived consequences of permanent ASF establishment

Consequences can be grouped into economic, food security, institutional and sociocultural dimensions. Across all sectors, participants described mutually reinforcing losses: producers anticipated the collapse of herd investment and restricted access to finance; government officials described the loss of tax revenue, export income, and employment; and hotel representatives noted that the interruption of the farm-feed pathway would substantially increase organic waste disposal costs. Participants connected ASF permanence to inflationary pressure on food prices, already observed after the 2021 outbreak, and highlighted that small-scale and backyard producers depend on pig keeping for household protein and income, making losses particularly acute in rural areas. Veterinary professionals warned that sustained outbreak response would exhaust services and crowd out surveillance of other diseases. Emotional and psychological costs (e.g., producer stress, repeated exposure to mass mortality, and loss of investment confidence) were also raised as important but undercounted consequences. Whilst ASF poses no direct zoonotic risk, participants noted that losses in consumer confidence and movement restrictions could indirectly affect regional tourism and demand for pork.

### The organic waste system

**Figure 1:**
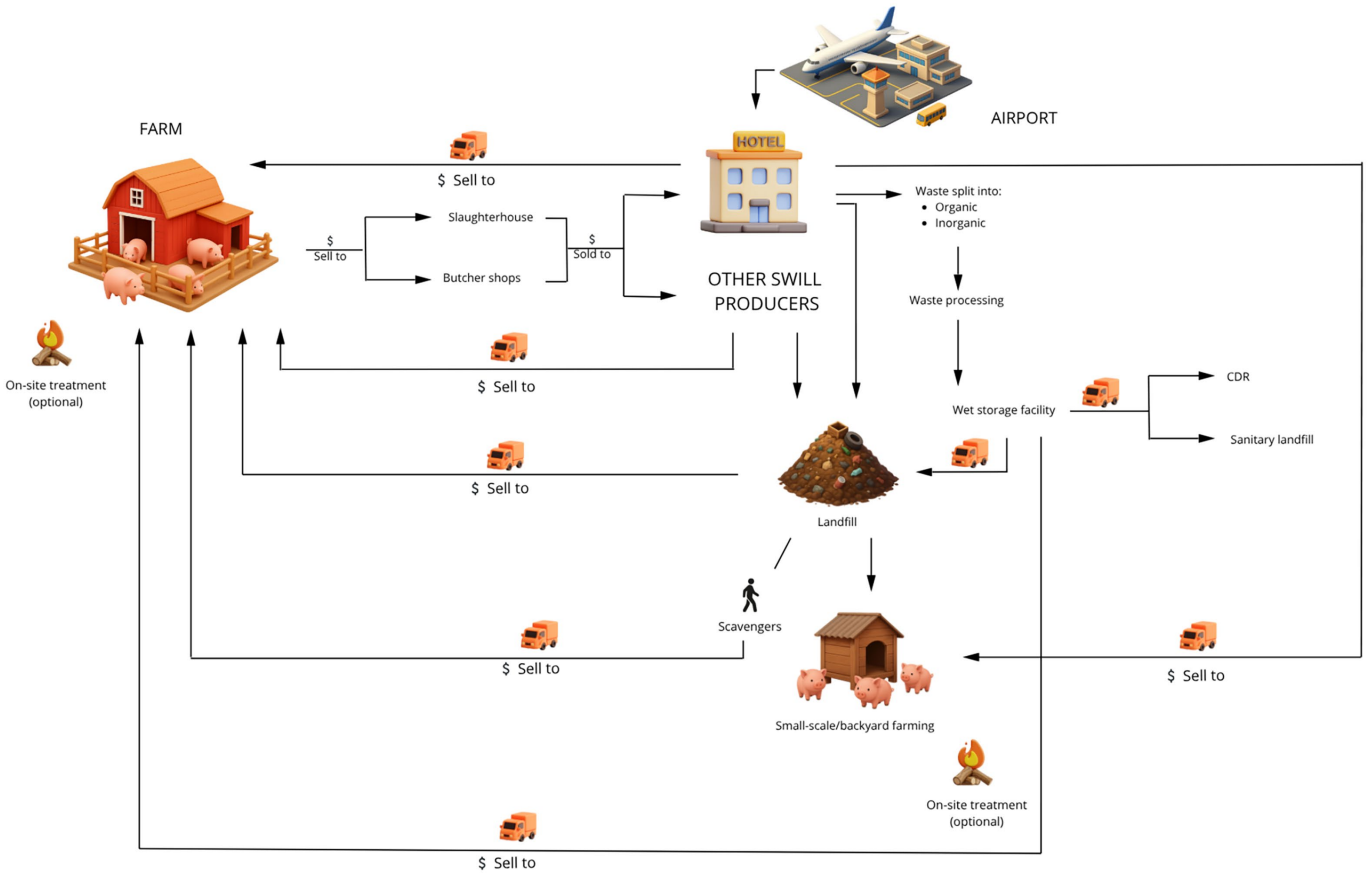
A composite of the waste management flowchart drawn by the five sectors participating in the workshop “Organic Waste Flow as an ASF Vulnerability: A Multi-Sector Participatory Systems Workshop in Eastern Dominican Republic” held in Santo Domingo, Dominican Republic, in February 2026. CDR: combustible derivado de residuos (waste-derived fuel).

System maps produced in mixed-sector tables revealed a multi-layered structure with formal and informal components (Figure 1). Hotels and resorts were identified as sources of large volumes of food waste, including vegetables, meat, and bakery products. Various collectors serve hotels in the region, ranging from formally registered companies with hotel contracts to informal operators who collect opportunistically. Economic relationships were bidirectional: most hotels paid collectors for the removal of their organic waste, whilst others may have received payment when the waste had value as animal feed. Workshop participants estimated that hotels in the eastern region generate between 1,000 and 2,000 kg of separated organic waste per day (31,000–61,000 kg/month).

Three primary destinations for organic waste were mapped consistently: municipal landfills, legal and illegal open dumps (*vertederos*), and, to a much lesser extent, composting. Excess organic waste was directed to *vertederos*, described as open, uncontrolled sites where free-roaming pigs, dogs, birds, and informal waste pickers or scavengers (“*buzos*”) continuously interact with organic material, creating a secondary exposure route. Informal, unrecorded flows, including off-contract pickups, direct hotel-to-producer arrangements, and illegal disposal of organic waste, were pervasive and structurally invisible to animal health surveillance systems. Participants estimated that about 5% of hotel waste goes to smallholder systems.

Green stickers clustered around formalised hotel–collector relationships and the existence of producer associations. Red stickers concentrated on irregular collection frequency, absent waste segregation at source (with organic waste routinely mixed with packaging, plastics and batteries), limited storage infrastructure, and the near total absence of heat treatment of organic waste before being offered to animals. These pain/friction points were consolidated into five transversal themes for Day 2: (a) heat treatment—inconsistent and unverifiable; (b) transport and traceability—informal and unrecorded; (c) regulatory implementation for organic waste disposal—norms exist, but enforcement is weak; (d) management of waste—defaults to *vertederos*; and (e) infrastructure—improve smallholders biosecurity.

### Sectoral perspectives

The normative group described national and municipal regulations restricting untreated swill feeding but reported that enforcement is rare and reactive, triggered by outbreaks rather than routine monitoring. Key barriers included insufficient inspection staff, a lack of inter-institutional coordination protocols amongst DIGEGA, the Ministry of Environment, and municipalities, and no systematic registry of collectors or farms. Participants converged on improved registration and permitting of collectors as the most feasible short-term regulatory step, however, acknowledging that enforcement has historically relied on sanctions rather than incentive mechanisms.

The productive chain group described an operational chain in which hotel waste-separation efforts (into non-organic and organic, with further separation into vegetables, meats, and bakery) are routinely undermined when waste is mixed during collection. Hotel representatives expressed there is growing interest in a certified “green chain” as a differentiating element for the company’s environmental, social and governance reporting. Collectors described economic pressure to maximise loads and minimise turnaround times, with no biosecurity protocols between pickups, creating a potential epidemiological bridge across multiple farms on a single route (e.g., leachate contaminating streets and other farms), usually using unregulated vehicles. Producers described food waste as an economically essential feed input, typically fed fresh without heat treatment, citing fuel costs, lack of equipment and legal uncertainty as barriers. They also identified sourcing replacement pigs from unregistered backyard operations that enter the formal hotel food production chain during periods of high demand as a separate, underappreciated ASF introduction risk.

### Stakeholder’s divergent views

Intra-sectoral differences emerged within municipal actors, where environmental officials cited limited personnel and budgets for waste enforcement, whilst municipal waste authorities emphasised legal frameworks and attributed issues to business behaviours. Amongst veterinarians/epidemiologists, views split on licenced transporters: some saw traceability benefits, others viewed multi-farm routes as contamination risks.

Inter-sectoral tensions were evident between hotels and municipalities. Hotels highlighted formal waste sales to licenced firms as a benefit, but officials noted informal diversions to farms despite regulations. These disagreements on the system were captured via independent group mapping followed by plenary synthesis, preserving divergent views as coexisting pathways rather than forcing consensus.

### Scenario analysis

Under Scenario 1 (no change), participants estimated a 10 to15-day detection lag for an ASF outbreak linked to organic waste, with backyard operations likely resolving mortality events informally before notification. Current investigation protocols do not trace waste provenance, so the swill pathway would not routinely be investigated even in reported outbreaks. Scenario 2 (low-cost improvements) was judged capable of meaningfully reducing the amount of virus-viable swill reaching farms, with centralised heat treatment at collection points identified as more practical than on-farm treatment. Concerns included cost allocation, the quality of organic waste segregation (e.g., heating organic waste that may contain plastic or alkaline batteries), logistical demands on smallholders, and biosecurity risks from increased worker movement. Scenario 3 (traceability and certification) would require support from producer associations (e.g., FEDOPOR, ADOGRANJA), DIGEGA’s veterinary capacity, the adoption of successful traceability experience in the Dominican Republic, and a functional culling compensation system. However, achieving scenario 3 relies on overcoming critical gaps: there is no national producer registry capturing backyard units, no waste-flow traceability, insufficient field staff, and no mechanism to reward compliant actors. Participants converged on the need to regulate the system rather than focus on individual actors (e.g., pig producers).

### STEEP barriers

The STEEP analysis identified social inertia, limited affordable heat-treatment equipment, misaligned cost–benefit distribution of improving the management and use of organic waste, environmental trade-offs from fuel-based cooking, and a legal prohibition on swill feeding that creates a grey area discouraging open disclosure as the principal barriers to change. A pragmatic licenced-swill model analogous to Puerto Rico’s was widely seen as more effective than attempting to enforce an outright ban, although it would only reduce the risk of ASF associated with swill consumption, not the risk associated with the movement of swill from the source to the production sites. A detail of the STEEP barriers explored is presented in Table 1.

**Table 1:** STEEP analysis of barriers to improve the management of organic waste used to feed pigs.

STEEP analysis					
Factors	Effective enforcement based on existing regulations	Compliance with regulations: animals in open dumps	Infrastructure: improvements amongst small-scale pig producers	Formalisation of waste collectors	Producers: thermal treatment of food waste
Social	Implementing effective enforcement of existing regulations related to waste management, biosecurity, and sanitary practises in pig production may face challenges when informal practises occur within inspection and oversight processes. In some situations, both the inspector and the regulated actor may benefit from avoiding the strict application of regulations, which makes consistent enforcement more difficult.	Compliance with regulations that restrict the presence of animals in open dumps may be difficult due to established practises and subsistence strategies. Some backyards or small-scale producers use food waste from open dumps as a low-cost feed source, which significantly reduces feeding costs. As a result, these producers may benefit economically from maintaining this practise, creating resistance to change.	Adopting infrastructure improvements amongst small-scale pig producers may face challenges due to established production habits, resistance to change, and traditional management practises. Additionally, limited access to education and technical training may restrict producers’ knowledge about biosecurity and infrastructure improvements. Under these conditions, pig producers do not benefit from maintaining current infrastructure, whilst producers of other livestock species may benefit if pig production remains less modernised or productive.	Formalising waste collectors may face resistance because it requires compliance with certification requirements, biosecurity protocols, and sanitary standards for the transport of organic waste. These requirements may require collectors to change their current work practises.	Adopting thermal treatment of food waste as a sanitary practise may face resistance because it requires transforming a technical procedure into a consistent habit. Some producers may initially adopt the practise but may not maintain it consistently over time.
Technological	Inspections do not require complex technologies, as the necessary technical knowledge for sanitary and environmental inspections already exists and is accessible to regulatory institutions. However, tools such as official producer registries, inspection databases, digital monitoring systems, and compliance reporting platforms could strengthen enforcement and monitoring processes.	The infrastructure needed to prevent animals from accessing open dumps already exists and is generally accessible. This may include perimeter fencing, waste containment systems, controlled open dumps access points, waste sorting infrastructure, and basic monitoring or surveillance mechanisms.	The equipment and technical knowledge required to improve production infrastructure already exist and are accessible, including biosecurity facilities, animal housing improvements, and cleaning and disinfection systems. Access to these improvements may be facilitated through loans from the Agricultural Bank or support from producer associations.	The equipment and technical knowledge required for this regulation already exist and are accessible. Veterinary services, national institutions such as DIGEGA, and international organisations such as FAO could provide training in biosecurity, safe waste handling, and sanitary transportation of organic waste. Required equipment may include appropriate transport vehicles, cleaning and disinfection systems, and personal protective equipment.	Boiling food waste is not a highly complex technique, but it may require equipment such as boilers, large cooking containers, heat-resistant vessels, and tools for mixing and handling the waste. Producers must also have the knowledge to inspect and sort waste, removing non-organic materials such as plastics or metals before boiling.
Economic	Strengthening regulatory enforcement would involve operational costs for government institutions, particularly the Ministry of Environment, including personnel, inspections, and monitoring activities. At the same time, enforcement may generate government revenue through administrative fines or penalties for regulatory non-compliance.	Using food waste from open dumps as animal feed helps reduce production costs, particularly for small-scale producers. Implementing measures to prevent this practise would require additional investment from municipal governments, which are responsible for open dumps management and infrastructure improvements.	Infrastructure improvements involve significant financial investment for producers. In some cases, financial resources obtained through support programmes may not be used efficiently. In the context of African Swine Fever (ASF), financial support programmes for pig production have been restricted. The National Biosecurity Commission provides credit primarily to producers who comply with biosecurity standards.	Compliance with certification requirements and biosecurity protocols may involve significant costs for collectors, including certification fees and the costs of cleaning and disinfecting vehicles used to transport waste.	Producers would bear the costs associated with adopting this practise. If boiling is carried out using gas or electricity, energy costs may increase significantly.
Environmental	If existing environmental regulations were enforced effectively, improvements in environmental conditions could occur, including reduced contamination of soil, water, and air associated with improper waste management and unregulated production practises.	Compliance with these regulations could generate environmental benefits, including reduced disease transmission, fewer odours, and lower contamination of soil, water, and air associated with organic waste and animal exposure to waste in open dumps.	Improving production infrastructure could generate environmental benefits, including improved manure storage and disposal systems, reduced runoff contamination affecting nearby soil and water sources, and stronger biosecurity practises that limit pathogen spread between farms, which could also contribute to improving the environmental perception and sustainability of the sector.	Formalising the sector could reduce the amount of organic waste sent to open dumps, preventing approximately 50–80% of this waste from contributing to environmental pollution and greenhouse gas emissions.	Using firewood as a fuel source for boiling waste may generate air pollution and increase pressure on forest resources due to wood extraction.
Political	This change can be implemented within the current legal framework, since the relevant regulations already exist. However, it would require clear enforcement mechanisms and a defined system of sanctions and consequences. The Ministry of Environment would play a central role in overseeing implementation and monitoring compliance.	This change can be implemented within the existing legal framework, as regulations already exist. However, stronger monitoring and enforcement mechanisms would be necessary. Municipal governments would play a key role in managing and controlling access to open dumps.	Implementing these changes may face limitations related to corruption, outdated regulations within the current legal framework (particularly Law 40–30), and weaknesses in enforcement mechanisms. Producer associations, with support from DIGEGA, could play an important role in facilitating implementation.	Implementation may face challenges if the State maintains strict certification requirements, certification renewal frequency, or high regulatory standards, which could make formalisation more difficult for collectors.	This practise is currently not compatible with the existing legal framework, specifically Decree 607–05 under Law 40–30, and implementing it would require a revision of the current legal framework.

### Impact/effort matrix

The impact/effort matrix produced three action clusters and a research agenda. Immediate actions that were seen as low-effort, high-impact were the enforcement of the norm that regulates “*vertederos*,” including access for people and animals to them. Short-term, low-effort priorities included waste segregation at hotel source, basic collector record-keeping, vehicle disinfection protocols and practical heat-treatment training for producers. Medium-term actions requiring inter-sector agreements included standardised hotel–collector contracts, formal collector registration and to pilot “green supply chain” (28) or provide incentives for the build-up of a sustainable product chain. Structural, longer-term actions included centralised heat-treatment infrastructure, transition from *vertederos* to sanitary landfills, regulatory reform to create a licenced-swill pathway, a national producer registry and economic incentive schemes for compliant actors. Unanswered questions identified in this workshop included the contribution of non-hotel waste sources (e.g., restaurants, “*fondas*,” schools), whilst quantitative flow data and the cost–benefit of treatment infrastructure for organic waste were identified as relevant to the Dominican Republic research agenda.

## Discussion

This workshop represents the first structured, multi-stakeholder analysis of the organic waste–swill–pig interface in the Dominican Republic, conducted 4 years into an endemic ASF situation with no clear path to elimination. Its key finding is that this organic waste system functions as a structurally hidden, multi-actor epidemiological risk network that no single institution comprehensively monitors. The system mapping revealed a multi-layered waste chain that extends and operationalises previous research on the value chain in the Dominican Republic. Hentz (16) observed that farmers often bring pigs to scavenging at open landfill dumps and feed raw food waste with minimal thermal treatment; Schambow et al. (11) found that 26% of ASF-affected producers, mainly backyard farms, suspected swill or garbage feeding as the source of the virus, with one farm citing proximity to a landfill. The FAO’s qualitative risk assessment for the Americas explicitly identified open landfills in Hispaniola as a major biosecurity risk and recommended fencing and regulation to prevent spreading (15). The widespread lack of heat treatment in the Dominican Republic’s organic waste system represents a major risk; Torok et al. (29) showed that heat treatment effectively destroys ASFV in swill, and the WOAH standard of 90 °C for 60 min with continuous stirring underpins Puerto Rico’s licenced programme discussed in the workshop. The main barrier in the Dominican Republic is not technical feasibility but the absence of a licencing system, economic incentives, and clear regulations that distinguish it from Puerto Rico and other successful models (29).

The normative group’s account of reactive, sanction-based enforcement aligns with broader epidemiological findings: Schambow et al. (11) observed that producers may infect their pigs when the compensation rate is higher than the market price or sell ASF-infected pigs to informal butcher shops (*carnicerias*) when market prices exceed government compensation rates (11), a behaviour also documented in other ASF-affected countries (30). The finding that hotel waste segregation is routinely compromised during collection (e.g., organic waste mixed with plastics and batteries) is critically important, as contaminated mixed waste complicates heat-treatment logistics, making upstream source segregation a necessary precondition. The productive chain group’s identification of sourcing replacement pigs from unregistered backyard operations as an underappreciated introduction risk aligns with Hentz (16), who found that herd scarcity after 2021 intensified this practise, with buyers travelling long distances to obtain animals. The workshop consensus that centralised heat treatment at collection points (Scenario 2) could be more practical than on-farm treatment is directly supported by the Japanese experience reported by Torok et al. (29), in which industrial plants operate with strict biosecurity zoning, providing certified feed at roughly half the cost of conventional compound feed. Scenario 3, which includes a national producer registry, veterinary field capacity, functional compensation, and traceability, aligns with the progressive control approach advocated by Schambow et al. (12), inspired by the Sardinia analogy and the foot-and-mouth disease progressive control pathway. Given that the ASF situation in the Dominican Republic aligns with endemicity rather than active epidemic growth, swift eradication remains unlikely without structural system change, and therefore incremental capacity-building is a more achievable pathway (11).

The STEEP analysis documented barriers with important local specificity. The social behaviour of using untreated food waste to feed pigs is regional: 19 of 35 countries surveyed in the Americas reported swill feeding as a common practise despite prohibitions (15). The economic misalignment, in which compliance costs fall on producers and collectors whilst enforcement benefits accrue to the government, is a structural disincentive that Schambow et al. (12) identified and recommended that biosecurity registration be accompanied by targeted infrastructure funding. The ecological observation that improved waste management could reduce landfill loading by 50–80% resonates with estimates that redirecting half of EU retail and catering surplus to pig feed would reduce greenhouse gas emissions by 5.8 million tonnes of CO_2_ equivalent per year, suggesting that environmental co-benefits could unlock financing beyond animal health budgets (29). Politically, the legal ban on swill feeding creates a grey area that makes inspection and practise improvement impossible. Jurisdictions that regulate rather than prohibit mandating heat treatment through licenced, certified programmes, as in Puerto Rico and Japan, achieve better biosecurity outcomes (29). The Impact/Effort Matrix identified *vertedero* access restriction as the highest-impact, lowest-effort priority as it requires no new legislation, no capital investment, and only coordination between municipal councils, the Ministry of Environment, and DIGEGA.

Despite the various perspectives represented in the workshop, various actors were identified as missing from the system maps. Waste collectors declined to participate, meaning their routes, load volumes, and biosecurity practises were reconstructed solely from observations in other sectors (Hotels at the top end of the chain, farmers at the receiving end of the supply chain) rather than from first-hand accounts. Beyond collectors, the workshop’s design centred on the hotel sector as the primary waste generator, but left restaurants, local *fondas* (family-run eateries), school canteens, and other food service establishments entirely unrepresented. These sources were identified as producers of *fregadura—*the liquid mixture of kitchen runoff, food scraps, cooking oils, and meat-contact wash water collected from food service sinks, which is a high-volume, informally circulated swill that operates through collection networks wholly separate from the hotel chain and with even less oversight or source segregation. Evidence from the Dominican Republic pig production system suggests food waste sourced from local commercial establishments, collected in large barrels and circulated through village networks, is already a documented feed input for smallholder producers, yet its contribution to the total organic waste reaching farms remains entirely unquantified. Thus, the system maps reported in this study focused on hotels and cannot be understood as fully representative of the whole organic waste system contributing to swine production in the region.

The participatory system-mapping workshop produced three substantive conclusions. First, the hotel–waste–pig farm circuit is a real and functioning pathway for organic waste in the eastern Dominican Republic, operating through both formal (licenced intermediary companies) and informal (unregulated diversion, landfill scavenging) channels. The coexistence of these channels substantially reduces traceability and increases the biosecurity risk of ASF transmission. Second, governance of this system is fragmented across at least four regulatory bodies—Medio Ambiente, municipal authorities (ayuntamientos), the General Livestock Directorate (DIGEGA), and the Ministry of Public Health—with overlapping but poorly coordinated mandates and insufficient enforcement capacity. Third, there is sectoral consensus that the hotel sector is the most tractable entry point for regulatory reform, given its existing formal waste-management practises, contractual relationships, and governance capacity, even though it accounts for only a small portion of the total organic waste stream reaching piggeries.

Based on these conclusions, we identify the following strategic lines for future work: (i) a prospective quantitative waste-tracking study to provide empirical estimates of the volume and composition of hotel-sourced waste entering the swine production system; (ii) a targeted survey of restaurants and *fondas*, which were excluded from this first study due to governance constraints, but which are likely a larger informal waste source; (iii) a regulatory mapping exercise to identify the specific legislative mechanisms that could be activated to require heat treatment at waste collection points, with Puerto Rico’s APHIS-administered permit system as a potential model; and (iv) a biosecurity capacity-building programme for smallholder pig producers in the Eastern region, given the evidence that farms practising higher biosecurity standards actively refuse food waste, whilst smallholder farms are disproportionately reliant on it. This agenda would help fill quantitative gaps left by previous epidemiological and value chain studies conducted in the Dominican Republic (11, 12, 16).

## Data Availability

The datasets presented in this article are not readily available because data is the result of a workshop conducted under Chatham House rules. Requests to access the datasets should be directed to juan.villanueva@uss.cl.

## References

[1] Williams DT Mettenleiter TC Blome S. African swine fever: advances and challenges. Rev Sci Tech. (2024):58–69. doi: 10.20506/rst.SE.3559, 39713833

[2] Nuanualsuwan S Songkasupa T Boonpornprasert P Suwankitwat N Lohlamoh W Nuengjamnong C. Thermal inactivation of African swine fever virus in swill. Front Vet Sci. (2022) 9:6064. doi: 10.3389/fvets.2022.906064, 35733638 PMC9207410

[3] Davies K Goatley LC Guinat C Netherton CL Gubbins S Dixon LK . Survival of African swine fever virus in excretions from pigs experimentally infected with the Georgia 2007/1 isolate. Transbound Emerg Dis. (2017) 64:425–31. doi: 10.1111/tbed.12381, 26104842 PMC5347838

[4] Chu X Ge S Zuo Y Cui J Sha Z Han N . Thoughts on the research of African swine fever live-attenuated vaccines. Vaccine. (2024) 42:126052. doi: 10.1016/j.vaccine.2024.06.020, 38906762

[5] de Menezes TC Countryman AM Pendell DL Rushton J Tickel J Simmons H. Potential economy-wide impacts of an African swine fever outbreak in the United States. Front Vet Sci. (2026) 13:1752899. doi: 10.3389/fvets.2026.1752899, 41704817 PMC12908596

[6] You S Liu T Zhang M Zhao X Dong Y Wu B . African swine fever outbreaks in China led to gross domestic product and economic losses. Nat Food. (2021) 2:802–8. doi: 10.1038/s43016-021-00362-1 37117973

[7] Chuong VD Schambow RA Diep NT Minh PQ Van Long N To Nga BT . Epidemiology and control of African swine fever in Vietnam: a scoping review. Pathogens. (2025) 14:329. doi: 10.3390/pathogens14040329, 40333166 PMC12030036

[8] Gonzales W Moreno C Duran U Henao N Bencosme M Lora P . African swine fever in the Dominican Republic. Transbound Emerg Dis. (2021) 68:3018–9. doi: 10.1111/tbed.14341, 34609795

[9] Endris RG Haslett TM Hess WR. Experimental transmission of African swine fever virus by the tick Ornithodoros (Alectorobius) puertoricensis (Acari: Argasidae). J Med Entomol. (1991) 28:854–8. doi: 10.1093/jmedent/28.6.854, 1770521

[10] Butler JF Gibbs EPJ. Distribution of potential soft tick vectors of African swine fever in the Caribbean region (Acari: Argasidae). Prev Vet Med. (1984) 2:63–70. doi: 10.1016/0167-5877(84)90049-7

[11] Schambow RA Hussain S Antognoli MC Kreindel S Reyes R Perez AM. Epidemiological assessment of African swine fever spread in the Dominican Republic. Pathogens. (2023) 12:1414. doi: 10.3390/pathogens12121414, 38133297 PMC10746036

[12] Schambow RA Carrasquillo N Kreindel S Perez AM. An update on active and passive surveillance for African swine fever in the Dominican Republic. Sci Rep. (2025) 15:2244. doi: 10.1038/s41598-025-86690-9, 39833369 PMC11747335

[13] Arias J. Inventario Nacional Porcino. 47 (Convenio Ministerio De Agricultura – OIRSA). Santo Domingo: Ministerio de Agricultura de Republica Dominicana (2022).

[14] FAO (2025). Available online at: https://www.fao.org/animal-health/news-events/news/detail/dominican-transporters-strengthen-swine-biosecurity-with-support-from-fao-and-the-national-government/en (Accessed March 30, 2026).

[15] Rozstalnyy A Roche X TagoPacheco D Kamata A BeltranAlcrudo D Khomenko S . Qualitative Risk Assessment for African Swine fever Virus Introduction. Rome: FAO Animal Production and Health Papers (2022).

[16] Hentz A. (2023). Mapping the Pig Value Chain in the Dominican Republic with Emphasis on the Risk of Transmission of African Swine Fever

[17] Zinsstag J Schelling E Crump L Whittaker M Tanner M Stephen C. One Health: The Theory and Practice of Integrated Health Approaches. 2nd ed. Wallingford: CAB International (2015).

[18] Chatham House, (n.d.). Chatham House Rule | Chatham House – International Affairs Think Tank. London: Chatham house. Available online at: https://www.chathamhouse.org/about-us/chatham-house-rule (Accessed March 16, 2026).

[19] Ministerio de Medio Ambiente y Recursos Naturales, (2026). Available online at: https://ambiente.gob.do/ (Accessed March 18, 2026).

[20] Hervé-Claude LP Gallagher C Navarrete-Talloni MJ Boden L Villanueva-Cabezas JP. African swine fever knowledge, attitudes and practices of pig farmers of saint Kitts, Nevis, saint Eustatius, Saint Lucia and Saba; West Indies. Front Vet Sci. (2025) 12:1710806. doi: 10.3389/fvets.2025.1710806, 41451337 PMC12729110

[21] Denzin NLY. The SAGE Handbook of Action Research: Participative Inquiry and Practice. London: Google eBook (2007). p. 752.

[22] Voinov A Bousquet F. Modelling with stakeholders. Environ Model Softw. (2010) 25:1268–81. doi: 10.1016/j.envsoft.2010.03.007

[23] Chambers R., (2012). Participatory Workshops. London: Earthscan.

[24] Littlewood KE Gardner DH. A brief guide to qualitative research in veterinary science: interviews, focus groups, surveys and reflexive thematic analysis for practitioners and researchers. N Z Vet J. (2026):1–11. doi: 10.1080/00480169.2026.2614562, 41628605

[25] Martín-López B Daw T Bohensky EL Butler J Hill R Martin-Ortega J . Participatory scenario planning in place-based social-ecological research: insights and experiences from 23 case studies. Ecol Soc. (2015) 20:32. doi: 10.5751/ES-07985-200432

[26] Aguilar FJ Francis J. Scanning the Business Environment. New York: Macmillan (1967).

[27] National Collaboration Centre for Methods and Tools, (n.d.). Impact Effort Matrix | Organizational Change Record. Hamilton, Ontario: National Collaboration Centre for Methods and Tools. Available online at: https://www.nccmt.ca/organizational-change/results/2 (Accessed March 16, 2026).

[28] Maditati DR Munim ZH Schramm HJ Kummer S. A review of green supply chain management: from bibliometric analysis to a conceptual framework and future research directions. Resour Conserv Recycl. (2018) 139:150–62. doi: 10.1016/j.resconrec.2018.08.004

[29] Torok VA Luyckx K Lapidge S. Human food waste to animal feed: opportunities and challenges. Anim Prod Sci. (2022) 62:1129–39. doi: 10.1071/AN20631

[30] Nguyen-Thi T Pham-Thi-Ngoc L Nguyen-Ngoc Q Dang-Xuan S Lee HS Nguyen-Viet H . An assessment of the economic impacts of the 2019 African swine fever outbreaks in Vietnam. Front. Vet. Sci. (2021) 8:686038. doi: 10.3389/fvets.2021.686038, 34760953 PMC8573105

